# CheloniansTraits: a comprehensive trait database of global turtles and tortoises

**DOI:** 10.1038/s41597-025-05089-3

**Published:** 2025-05-22

**Authors:** Jiang Wang, Yunhao Xu, Huiwen Zhu, Chuanwu Chen, Yifan Zhao, Yanping Wang

**Affiliations:** Laboratory of Island Biogeography and Conservation Biology, College of Life Sciences, Nanjing Normal University, Nanjing, 210023 China

**Keywords:** Biodiversity, Conservation biology, Biogeography

## Abstract

Turtles and tortoises (chelonians) possess a variety of ecological characteristics, including long lifespans and protective shells, which have enabled them to survive and adapt to environmental challenges since the Triassic period. However, many characteristics of chelonians have turned into disadvantages for their populations in the Anthropocene. Currently, there remains a lack of comprehensive data on the morphological, life-history, and ecological characteristics of all chelonians on a global scale. Consequently, our study aims to collect a complete trait database of global chelonians (CheloniansTraits), which may help bridge the knowledge gap regarding the identity and ecology of global chelonians and thereby aiding future conservation endeavors. We compiled 69 trait data for all 358 recognized chelonian species, utilizing ~2,000 literature sources, covering 33 morphological, 21 life-history, 7 ecological traits, and 8 conservation information. This database serves as a uniquely valuable resource for exploring evolutionary, biogeographical, and ecological inquiries related to chelonians, as well as elucidating key aspects of ecological strategy variation among species.

## Background & Summary

Turtles and tortoises, or chelonians (Order: Testudines), tracing back to the Triassic period, have an older origin compared to other modern vertebrate clades^1,2^. Chelonians are comprised of more than 300 species that inhabit various aquatic and terrestrial ecosystems and are widespread worldwide, except Antarctica^3,4^. Over 200 million years, chelonians have developed a variety of unique morphological, life-history, and ecological traits that have enabled them to survive and adapt to environmental challenges^5^. They are also widely known for their ecological importance as mesopredators of animals and seed dispersers in ecosystems^5,6^.

In the Anthropocene, however, these unique traits have turned into disadvantages for Chelonian populations^7–9^. For instance, the shell, as an adaptive trait, provides shelter from the environment, shields against predators, enhances thermoregulation and serves as a valuable reservoir of fats, minerals, and water^10,11^. Nevertheless, the shelled-body restricts their mobility, especially terrestrial ones, making them easier to be captured and overexploited by humans^12,13^. Moreover, life-history traits such as long lifespan, delayed sexual maturity, and long generation times confer advantages for the survival of chelonians under long-term natural selection pressures^1,14,15^. Unfortunately, their long lifespan has also led many people to believe that consuming their meat and eggs or using them in traditional medicines will improve longevity^16–18^. Meanwhile, individuals with large body sizes and mature females are often sources of protein for local residents and the main targets of trade (e.g., in Amazonian cultures)^9,19^. Thus, the delayed sexual maturity and long generation times traits render them more difficult to recover from population losses^9,17^. In addition, chelonians have developed various abilities to adjust to extreme environmental conditions (e.g., thermal tolerance, resistance to food shortage, and anoxia tolerance), which make them withstand long-distance transportation in global trade^20,21^. In this context, populations of chelonians are declining rapidly and many species have gone extinct due to human activities^4,8^. For example, over half species of chelonians are threatened to extinction, as assessed by the International Union for Conservation of Nature (IUCN) Red List^22^. To date, chelonians rank among the most threatened vertebrate groups, second only to primates^9,23,24^.

Understanding the species traits of chelonians is vital for developing effective conservation strategies^25–27^. However, the database on the morphological, life-history, and ecological traits of chelonians is generally lacking compared to other vertebrate groups, such as mammals, birds, and amphibians^28–32^. In particular, the identification of chelonians largely relies on their morphological characteristics, while it remains a big challenge for customs and forestry law enforcement personnel regarding the complexities of these features. Despite that several turtle identification guides have been published, many of them lack effective identification guidance for non-experts to accurately judge and identify turtles. Furthermore, previous studies that compiled body size datasets, such as Itescu *et al*.^33^ and Regis and Meik^34^, have certain limitations. These limitations stem from insufficient representativeness due to a small number of species examined and traits included or the use of generalized size approximations rather than more precise size data. Aside from these compilations of turtle body size datasets, Colston *et al*.^35^ and Rodríguez-Caro *et al*.^36^ also published two datasets on the ecological or life-history traits of turtles. However, as summarized by Colston *et al*.^35^ and Rodríguez-Caro *et al*.^36^, these data are scarce and contain many missing values. While we acknowledge that past trait databases can be important and have laid the foundation for the availability of datasets, compiling a global dataset on chelonians will help drive the conservation of these species in regional or global contexts. The most recent trait database related to chelonians is ReptTraits^37^, but it only focused on the ecological traits of chelonians, lacking specificity and practicality for general use. Moreover, the taxonomic checklist of the ReptTraits^37^ is a little bit confusing, which includes subspecies and subpopulations of chelonians (e.g., *Elseya caelatus*, *Chelodina ipudinapi*, *Elseya auramemoria*). The ReptTraits also unnecessarily included some traits unsuitable for chelonians, such as venomous, venomous fangs, and reproductive mode^37^. In fact, after excluding these trait information, only eight (since maximum Longevity is also misclassified) morphological traits of chelonians were collected in the ReptTraits database. Finally, the ReptTraits dataset has poor data completeness on all the life-history traits (<40%), except for the body size (actually, the maximum body mass data primarily cited is converted data based on allometry sourced from Meiri *et al*.^38^) and the geographical distribution of chelonians^32^. Therefore, the ReptTraits dataset is inadequate to represent the full appearance of chelonians and also impractical for species conservation.

To bridge the gap, we collected a complete trait database of global chelonians (CheloniansTraits) that encompasses morphological traits, life-history traits, ecological traits, and conservation information. We made it possible by three-year efforts on collecting trait information from ~2,000 literature spanning from 1758 to 2024, related academic books such as “Turtles of the World: A Guide to Every Family”^39^ and “The Conservation Biology of Tortoises”^40^, existing morphological and life-history databases (e.g., Itescu *et al*.^33^, Regis & Meik^34^, Colston *et al*.^35^), the IUCN Red List (version 2024-2)^22^, the Reptile Database^41^. Our trait database was double-checked by specialists in this field to ensure completeness and accuracy. Overall, 358 currently recognized species (including the few that are known to have gone extinct in recent centuries) belonging to 14 families were checked with species-level identities. For these species, we collected 69 traits, including 33 morphological traits, 21 life-history traits, 7 ecological traits, and 8 variables related to conservation information. We aim to enrich our understanding of global chelonians through this trait dataset. We hope that our dataset can contribute to the development of studies and efforts on effective species conservation of chelonians.

## Methods

### Species checklist

We obtained the species-level taxonomy of chelonians based on the taxonomic checklist of the Turtle Taxonomy Working Group (357 species)^42^, the Reptile Database^41^, and a review of scientific literature sources spanning the period from 1758 to 2024. Some species that require further evidence for their species-level identity were categorized as controversial species. Finally, we included 358 chelonian species with confidently identified records in our study. For each species, we recorded the information on species order, family, genus, binomial species name, English name, and year of description from the Turtle Taxonomy Working Group^42^.

### Data extraction procedure

**Fig. 1:**
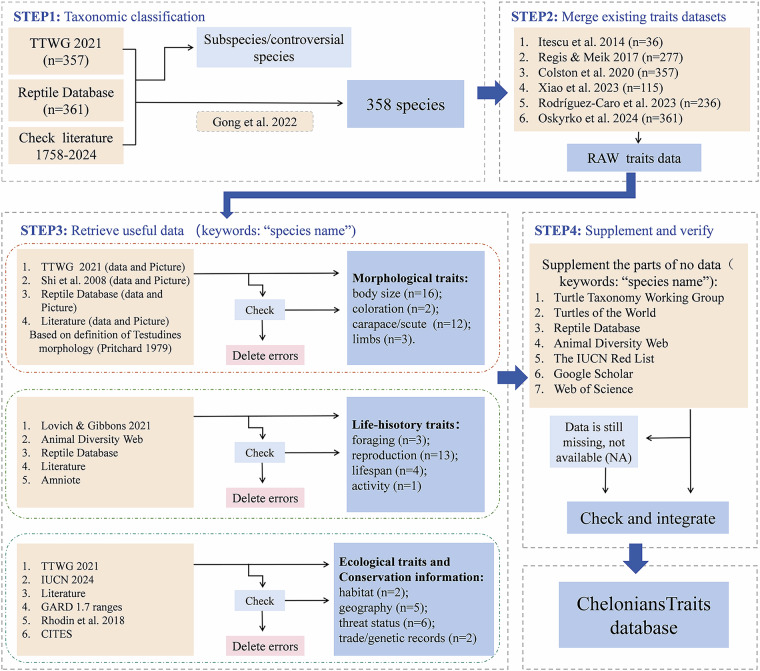
Elucidation of the workflow for dataset creation of global turtles and tortoises.

To compile a comprehensive trait dataset for chelonians, based on the initial survey, we selected 33 morphological traits, 21 life-history traits, 7 ecological traits, and 8 conservation information related to chelonians. These trait data reflect a variety of the unique morphological, ecological strategies, and functional roles of chelonians. They are primarily derived from extensive classification literature, academic books, and life-history databases^22,41–43^. Our data collection process primarily adheres to the following four steps (Fig. 1): (1) First, we confirmed 358 turtles and tortoises at the species-level based on the taxonomic checklist of the Turtle Taxonomy Working Group^42^, the Reptile Database^41^ and scientific literature sources (from 1758 to 2024). (2) Second, we integrated data from six primary datasets (Itescu *et al*.^33^, Regis & Meik^34^, Colston *et al*.^35^, Xiao *et al*.^44^, Rodríguez-Caro *et al*.^36^, and Oskyrko *et al*.^37^), compiling the raw data. (3) For morphological, life-history, ecological traits, and conservation information, we supplemented them by consulting literature^43,45,46^ and using dedicated websites^22,41,46^. We then checked raw data and newly-discovered species and deleted errors. (4) Finally, we supplemented missing data by searching the keyword ‘species name’, ‘turtles’, ‘morphological’, ‘reproductive’, ‘diet’, ‘active pattern’, and ‘clutch size’, on the Reptile Database (http://www.reptile-database.org)^41^, Animal Diversity Web (https://animaldiversity.org)^46^, the IUCN Red List (https://www.iucnredlist.org)^22^, Web of Science (https://www.webofscience.com), and Google Scholar (https://scholar.google.com) and integrated the chelonians traits database. In cases of data conflicts, we collected various values including maximum, mean and range data for parameters such as body size, lifespan, clutch size, number of clutches per year, incubation period, and egg size as much as possible. However, the data of diet or activity time can vary due to life stages, observations or seasonal changes^39,47,48^. Therefore, we extracted these values for individual traits by choosing data for the adult stage or main active patterns in our study. For example, green turtles (*Chelonia mydas*) are omnivorous during some life stages, ranging from carnivorous on marine animals as juveniles to becoming mostly herbivorous on marine plants as adults^39^. We recorded adult green turtles eating sea grass and macroalgae in the detailed diet data, categorizing their diet as herbivorous. We divided the activity patterns of turtles according to the timing of key behaviors such as foraging, basking, nesting, and movement mentioned in the primary source. The activity patterns of Blanding’s turtles (*Emys blandingii*) were described as diurnal because rarely recorded Blanding’s turtles moving at night^49^.

### Trait data

**Fig. 2:**
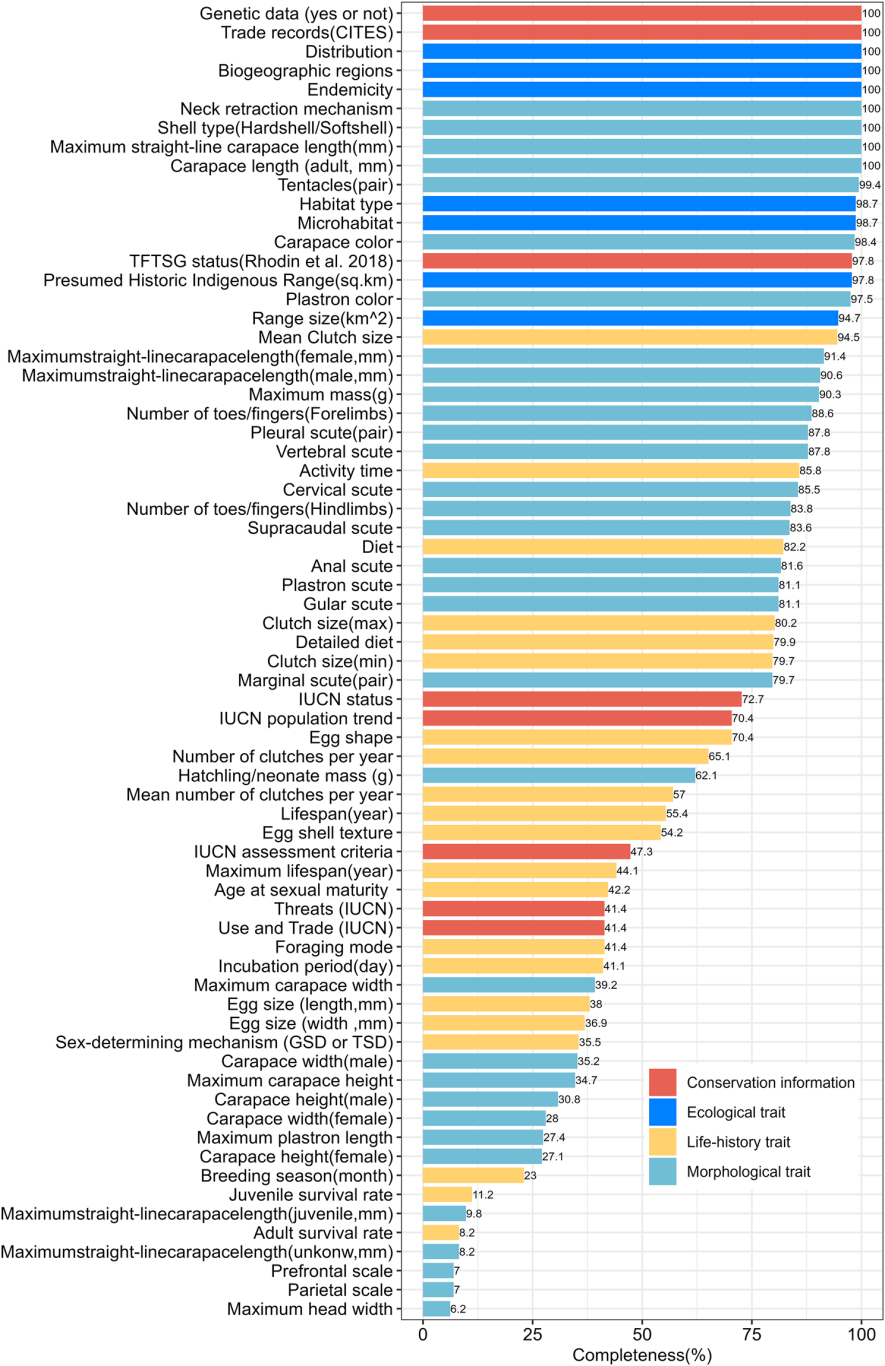
Percentage of data completeness for each trait released in the database.

For the data of 16 body size traits, we collected the carapace and plastron length data separately for adults, males, females, juveniles, and unknown-gender individuals. The data of head length and width for adults, maximum mass, and hatchling/neonate mass are also aggregated (Fig. 2). When multiple resources are available for a species, we prioritized the resource that contains the most extensive and comprehensive feature descriptions (e.g., TTWG 2021^42^). However, body size values are typically population-dependent, varying geographically, latitudinally, and altitudinally (e.g., *Testudo hermanni*)^42,50^. They are influenced by sample sizes and potentially biased collection methodologies^42^. We therefore also included ranges in the parameters to compare against and provide a comprehensive database. Non-continuous morphological traits, such as scutes, tentacles (pair), and number of toes/fingers in the forelimbs and hindlimbs, are often incompletely or inadequately documented in published literature or books. Therefore, we collected these data by referencing the descriptions of scales and scutes provided by Pritchard^51^ and using high-quality pictures from sources such as The Reptile Database^41^ and TTWG^42^. Specifically, approximately 90% of the non-continuous morphological trait measurements were derived from images provided by TTWG^42^.

Our dataset also includes 21 life-history traits. Turtles and tortoises have diverse diets, foraging modes, and activity time^37,39,49^. The long lifespan of chelonians is a unique life-history trait^9,39^. In addition to collecting the recorded lifespan of each species (in years), we have compiled details on their maximum lifespan (in years) as well. The adult survival rate and juvenile survival rate, which are associated with survival, are calculated as the arithmetic mean of survival values for specific life stages^36^.

We also gathered the reproductive traits of chelonians from literature descriptions^46,52,53^, including age at sexual maturity, breeding season (in months), incubation period (in days), clutch size (reported as minimum, maximum, and average values), number of clutches per year, egg size, eggshell texture and shape, and sex-determination systems.

For ecological traits, such as range sizes (km^2^), microhabitat, habitat type, endemicity, biogeographic regions, distribution, and presumed Historic Indigenous Range (km^2^), we reported these traits based on numerous previous studies^35,42,54^.

For conservation information, we obtained the threat status, assessment criteria, population trends, threats, use, and trade from the IUCN^22^. The threat status of chelonians, as assessed by Rhodin *et al*.^45^, was also included. Trade records spanning the period from 1758 to 2023 were collected from the Convention on International Trade in Endangered Species of Wild Fauna and Flora (CITES, https://trade.cites.org/)^55^, and the presence or absence of trade records (yes or no) for each species was also reported. Finally, we examined publicly available genetic data (GenBank, https://www.ncbi.nlm.nih.gov/)^56^ for each species and reported whether genetic data was accessible or not (yes or no).

## Data Records

The dataset is open access and available at the Figshare repository as ‘CheloniansTraits dataset.xlsx'^57^. We created an Excel spreadsheet that includes both the dataset content and the column descriptions as separate worksheets (Table 1: CheloniansTraits data, Table 2: Main sources of trait value, Table 3: References, Table 4: Trait definitions and the completeness level, Table 5: Controversial species, Table 6: Type specimen, Table 7: Source of images)^57^. We provided the most comprehensive trait database of chelonians to date, encompassing 358 species from 14 families. In total, we extracted and collected the data from 2,000 literature sources and 20 databases. In addition to species order, family, genus, binomial species name, English name, and year of description, our dataset includes 69 traits. The trait completeness varied from 6.20% to 100%, with higher completeness of ecological traits and conservation variables, followed by morphological traits and life-history traits (Fig. 2).

## Technical Validation

We implemented four steps to compile CheloniansTraits (Fig. 1) and meticulously reviewed the dataset to ensure consistency in variables. All data was manually gathered from the respective sources for each species and carefully recorded. To ensure traceability of the data, we incorporated both primary and secondary sources, clearly indicating their respective origins. Data sources that were difficult to interpret and lacked extractable raw data were excluded from the dataset. In addition, double control procedures were implemented to ensure the accuracy of the dataset. We confirmed and re-examined whether the problem data originated from transcription errors by cross-referencing the raw file with the source data. During the double-checking process, we verified the validity using multiple literature sources and made corrections or removed the data from the database as needed.

## Usage Notes

Our database is the most comprehensive trait data available to date that has a full species coverage, detailed morphological traits for the first time, and a standardized referencing index. Notably, CheloniansTraits exhibited a high coverage of traits for each species, surpassing previous datasets for reptiles or chelonians. This database serves as a uniquely valuable resource for exploring evolutionary, biogeographical, and ecological inquiries related to chelonians, as well as elucidating key aspects of ecological strategy variation among species.

## Data Availability

No custom code was used to generate the described databases.w

## References

[1] Lyson, T. R. *et al*. Fossorial origin of the turtle shell. *Current Biology* **26**, 1887–1894 (2016). 27426515 10.1016/j.cub.2016.05.020

[2] Shaffer, H. B., McCartney-Melstad, E., Near, T. J., Mount, G. G. & Spinks, P. Q. Phylogenomic analyses of 539 highly informative loci dates a fully resolved time tree for the major clades of living turtles (Testudines). *Molecular Phylogenetics and Evolution* **115**, 7–15 (2017). 28711671 10.1016/j.ympev.2017.07.006

[3] Angielczyk, K. D., Burroughs, R. W. & Feldman, C. R. Do turtles follow the rules? Latitudinal gradients in species richness, body size, and geographic range area of the world’s turtles. *Journal of Experimental Zoology Part B: Molecular and Developmental Evolution* **324**, 270–294 (2015). 25588662 10.1002/jez.b.22602

[4] Lovich, J. E., Ennen, J. R., Agha, M. & Gibbons, J. W. Where have all the turtles gone, and why does it matter? *BioScience* **68**, 771–791 (2018).

[5] Gibbons, J. W. Why do turtles live so long? *BioScience* **37**, 262–269 (1987).

[6] Blake, S. *et al*. Seed dispersal by Galápagos tortoises. *Journal of Biogeography* **39**, 1961–1972 (2012).

[7] Heppell, S. S. Application of life-history theory and population model analysis to turtle conservation. *Copeia* **1998**, 367–375 (1998).

[8] Lutcavage, M. E. Human impacts on sea turtle survival. In Wyneken, J., Lohmann, K. J. & Musick J. A. (Eds.). *The Biology of Sea Turtles* Volume I, 387–409pp. (CRC Press, 2017).

[9] Stanford, C. B. *et al*. Turtles and tortoises are in trouble. *Current Biology* **30**, R721–R735 (2020). 32574638 10.1016/j.cub.2020.04.088

[10] Zangerl, R. The turtle shell. In Gans, C., Bellairs, A.d’A. & Parsons, T. S. (eds). *Biology of the Reptilia* Vol. 1 Morphology A, 311–339. (Academic Press, London, 1969).

[11] Cherepanov, G. O. Nature of the turtle shell: morphogenetic causes of bone variability and its evolutionary implication. *Paleontological Journal* **50**, 1641–1648 (2016).

[12] Landberg, T., Mailhot, J. D. & Brainerd, E. L. Lung ventilation during treadmill locomotion in a terrestrial turtle, *Terrapene carolina*. *Journal of Experimental Biology* **206**, 3391–3404 (2003). 12939371 10.1242/jeb.00553

[13] Magwene, P. M. & Socha, J. J. Biomechanics of turtle shells: how whole shells fail in compression. *Journal of Experimental Zoology Part A: Ecological Genetics and Physiology* **319**, 86–98 (2013). 23203474 10.1002/jez.1773

[14] Congdon, J. D., Dunham, A. E. & van Loben Sels, R. C. Delayed sexual maturity and demographics of Blanding’s turtles (*Emydoidea blandingii*): implications for conservation and management of long‐lived organisms. *Conservation Biology* **7**, 826–833 (1993).

[15] Congdon, J. D., Reding, D. M. & Bronikowski, A. M. Testing hypotheses of aging in long-lived painted turtles (*Chrysemys picta*). *Experimental Gerontology* **38**, 765–772 (2003). 12855285 10.1016/s0531-5565(03)00106-2

[16] Lau, M. & Shi, H. T. Conservation and trade of terrestrial and freshwater turtles and tortoises in the People’s Republic of China. *Chelonian Research Monographs* **2**, 30–38 (2000).

[17] Eisemberg, C. C., Rose, M., Yaru, B. & Georges, A. Demonstrating decline of an iconic species under sustained indigenous harvest — the pig-nosed turtle (*Carettochelys insculpta*) in Papua New Guinea. *Biological Conservation* **144**, 2282–2288 (2011).

[18] Sigouin, A. *et al*. Priorities for the trade of less charismatic freshwater turtle and tortoise species. *Journal of Applied Ecology* **54**, 345–350 (2017).

[19] Schneider, L., Ferrara, C. R., Vogt, R. C. & Schaffer, C. Subsistence-level chelonian exploitation on the Rio Negro and one viable alternative. *Chelonian Conservation and Biology* **15**, 36–42 (2016).

[20] Krivoruchko, A. & Storey, K. B. Turtle anoxia tolerance: biochemistry and gene regulation. *Biochimica et Biophysica Acta (BBA)-General Subjects* **1850**, 1188–1196 (2015). 25662819 10.1016/j.bbagen.2015.02.001

[21] Luiselli, L., Starita, A., Carpaneto, G. M., Segniagbeto, G. H. & Amori, G. A short review of the international trade of wild tortoises and freshwater turtles across the world and throughout two decades. *Chelonian Conservation and Biology* **15**, 167–172 (2016).

[22] IUCN. The IUCN Red List of Threatened Species. https://www.iucnredlist.org (2024).

[23] Hoffmann, M. *et al*. The impact of conservation on the status of the world’s vertebrates. *Science* **330**, 1503–1509 (2010). 20978281 10.1126/science.1194442

[24] Cox, N. *et al*. A global reptile assessment highlights shared conservation needs of tetrapods. *Nature* **605**, 285–290 (2022). 35477765 10.1038/s41586-022-04664-7 PMC9095493

[25] Cadotte, M. W. The new diversity: management gains through insights into the functional diversity of communities. *Journal of Applied Ecology* **48**, 1067–1069 (2011).

[26] Devictor, V. *et al*. Spatial mismatch and congruence between taxonomic, phylogenetic and functional diversity: the need for integrative conservation strategies in a changing world. *Ecology Letters* **13**, 1030–1040 (2010). 20545736 10.1111/j.1461-0248.2010.01493.x

[27] Tilman, D. *et al*. Future threats to biodiversity and pathways to their prevention. *Nature* **546**, 73–81 (2017). 28569796 10.1038/nature22900

[28] Jones, K. E. *et al*. PanTHERIA: a species‐level database of life history, ecology, and geography of extant and recently extinct mammals. *Ecology* **90**, 2648 (2009).

[29] Myhrvold, N. P. *et al*. An amniote life‐history database to perform comparative analyses with birds, mammals, and reptiles. *Ecology* **96**, 3109 (2015).

[30] Vancine, M. H. *et al*. ATLANTIC AMPHIBIANS: a data set of amphibian communities from the Atlantic Forests of South America. *Ecology* **99**, 1692 (2018). 29953585 10.1002/ecy.2392

[31] Wang, Y. *et al*. A dataset on the life-history and ecological traits of Chinese birds. *Biodiversity Science* **29**, 1149–1153 (2021).

[32] Meiri, S. SquamBase—A database of squamate (Reptilia: Squamata) traits. *Global Ecology and Biogeography* **33**, e13812 (2024).

[33] Itescu, Y., Karraker, N. E., Raia, P., Pritchard, P. C. & Meiri, S. Is the island rule general? Turtles disagree. *Global Ecology and Biogeography* **23**, 689–700 (2014).

[34] Regis, K. W. & Meik, J. M. Allometry of sexual size dimorphism in turtles: a comparison of mass and length data. *PeerJ* **5**, e2914 (2017). 28149687 10.7717/peerj.2914 PMC5267567

[35] Colston, T. J., Kulkarni, P., Jetz, W. & Pyron, R. A. Phylogenetic and spatial distribution of evolutionary diversification, isolation, and threat in turtles and crocodilians (non-avian archosauromorphs). *BMC Evolutionary Biology* **20**, 1–16 (2020). 32650718 10.1186/s12862-020-01642-3 PMC7350713

[36] Rodríguez-Caro, R. C. *et al*. Anthropogenic impacts on threatened species erode functional diversity in chelonians and crocodilians. *Nature Communications* **14**, 1542 (2023). 36977697 10.1038/s41467-023-37089-5 PMC10050202

[37] Oskyrko, O., Mi, C., Meiri, S. & Du, W. ReptTraits: a comprehensive dataset of ecological traits in reptiles. *Scientific Data* **11**, 243 (2024). 38413613 10.1038/s41597-024-03079-5 PMC10899194

[38] Meiri, S. *et al*. Different solutions lead to similar life history traits across the great divides of the amniote tree of life. *Journal of Biological Research-Thessaloniki* **28**, 1–17 (2021). 10.1186/s40709-021-00134-9 PMC7869468 33557958

[39] Lovich, J. E. & Gibbons, W. *Turtles of the world: A guide to every family*. (Princeton University Press, New Jersey, 2021).

[40] Swingland, I. R. & Klemens, M. W. *The conservation biology of tortoises* Vol. 5 (IUCN, 1989).

[41] Uetz, P. The reptile database. https://reptile-database.reptarium.cz/ (2024).

[42] Turtle Taxonomy Working Group (TTWG). (Rhodin, A. G. K., *et al*). Turtles of the World Annotated Checklist and Atlas of Taxonomy, Synonymy, Distribution, and Conservation Status (9th Ed.). *Chelonian Research Monographs* **8**, 1–472 (2021).

[43] Gong, S., Fritz, U., Vamberger, M., Gao, Y. & Farkas, B. Disentangling the *Pelodiscus axenaria* complex, with the description of a new Chinese species and neotype designation for *P. axenaria* (Zhou, Zhang & Fang, 1991). *Zootaxa* **5125**, 131–143 (2022). 36101223 10.11646/zootaxa.5125.2.2

[44] Xiao, F., Lin, Z., Wang, J. & Shi, H. T. Shell shape-habitat correlations in extant turtles: A global-scale analysis. *Global Ecology and Conservation* **46**, e02543 (2023).

[45] Rhodin, A. G. *et al*. Global conservation status of turtles and tortoises (order Testudines). *Chelonian Conservation and Biology* **17**, 135–161 (2018).

[46] Myers, P. *et al*. The Animal Diversity Web (online). https://animaldiversity.org (2023).

[47] Smith, G. R. & Iverson, J. B. Diel activity patterns of the turtle assemblage of a northern Indiana lake. *The American Midland Naturalist* **152**, 156–164 (2004).

[48] Butterfield, T. G., Scoville, A., García, A. & Beck, D. D. Habitat use and activity patterns of a terrestrial turtle (*Rhinoclemmys rubida perixantha*) in a seasonally dry tropical forest. *Herpetologica* **74**, 226–235 (2018).

[49] Hjort Toms, A., Browning, L. V. T., Paterson, J. E., Angoh, S. Y. J. & Davy, C. M. Night moves: nocturnal movements of endangered spotted turtles and Blanding’s turtles. *Journal of Zoology* **316**, 40–48 (2022).

[50] Djordjevic, S. *et al*. Sexual body size and body shape dimorphism of *Testudo hermanni* in central and eastern Serbia. *Amphibia-Reptilia* **32**, 445–458 (2011).

[51] Pritchard, P. C. H. *Encyclopedia of turtles*. (TFH Publications, New Jersey, 1979).

[52] Georges, A., Guarino, F. & Bito, B. Freshwater turtles of the TransFly region of Papua New Guinea: notes on diversity, distribution, reproduction, harvest and trade. *Wildlife Research* **33**, 373–384 (2006).

[53] Nemesházi, E. & Bókony, V. HerpSexDet: the herpetological database of sex determination and sex reversal. *Scientific Data* **10**, 377 (2023). 37311775 10.1038/s41597-023-02268-y PMC10264413

[54] Olson, D. M. *et al*. Terrestrial ecoregions of the world: a new map of life on earth. *BioScience* **51**, 933–938 (2001).

[55] *CITES Trade Database* https://trade.cites.org/ (2024).

[56] *NCBI Sequence Read Archive* https://www.ncbi.nlm.nih.gov/ (2024).

[57] Wang, J. *et al*. CheloniansTraits: a comprehensive trait database of global turtles and tortoises, *figshare*, 10.6084/m9.figshare.28828241 (2025). 10.1038/s41597-025-05089-3 PMC12098739 40404775

